# P02.189. GOALS: bundled services to reduce the length of hospital stay in women undergoing gynecology oncology surgery

**DOI:** 10.1186/1472-6882-12-S1-P245

**Published:** 2012-06-12

**Authors:** J Mrachek, J Dusek, D Trebesch, S Sendelbach, J Haupt

**Affiliations:** 1 Northwest Anesthesia, Minneapolis, USA; 2 Allina Health System, Minneapolis, USA

## Purpose

To examine if a multi-modal integrative intervention (education, early feeding/activity, epidural, integrative therapies) impacts length of hospital stay (LOS) compared to historical controls in women undergoing surgery for known or suspected endometrial, ovarian, or cervical cancer.

## Methods

The multi-modal intervention combines various therapies into a cohesive program. Prior to surgery, participants receive explicit patient education, acupuncture and mind/body therapies to assist with physical and mental preparation for surgery. On the day of surgery, an epidural is placed prior to general anesthesia administration to manage post-operative pain. Patients undergo standard of care surgery. The evening after surgery, patients eat a high protein “surgical soft” dinner and nurses assist patients in walking down the hospital halls. Acupuncture is provided on post-operative days one and two for post-surgical pain relief, nausea control and for stimulation of bowel function. Patients’ electronic medical records are reviewed to obtain intra-operative and post-operative details including amount of medications administered, length of surgery, time of admission to post-anesthesia care unit, time of first ambulation out of the room, percent of diet eaten first 24 hours, nausea/pain level, discharge date/time/disposition, and patient satisfaction scores. The primary endpoint is the difference in patient LOS following surgery relative to a historical control. Secondary endpoints include FACT-G, State-Trait Anxiety Inventory, Patient Activated Measure before admission and roughly six months after discharge from the hospital.

## Results

Forty-one women were enrolled to date. The preliminary analysis indicated an average LOS of 2.57 days as compared to 2.89 to 3.39 days for historical controls. Final analyses will be presented at the meeting.

## Conclusion

A bundled approach to care appears to reduce hospital LOS in women having surgery for endometrial, ovarian, or cervical cancer. Further research is needed to examine the efficacy of this approach in a controlled clinical trial.

